# Helping Educators Learn Pediatric Pain Assessment and Intervention Needs Program (HELP PAIN): Program Development with Community Partners

**DOI:** 10.3390/children11111318

**Published:** 2024-10-30

**Authors:** Natoshia R. Cunningham, Michelle Adler, Jocelyn Zuckerman, Mallet R. Reid, Sarah C. Love, Kelly Theaker, Steven J. Pierce, Rachel Vandenbrink, Jeanne Paque, Andrea L. Wendling, Judith Arnetz

**Affiliations:** 1 Department of Family Medicine, Michigan State University, East Lansing, MI 48824, USA; maadler3@wisc.edu (M.A.); zucker32@msu.edu (J.Z.);; 2 College of Human Medicine, Michigan State University, Grand Rapids, MI 49503, USA; 3 Akron Children’s Hospital, Akron, OH 44308, USA; 4 Rockford Public Schools (Michigan), Rockford, MI 49341, USA; 5 Center for Statistical Training and Consulting, Michigan State University, East Lansing, MI 48824, USA; pierces1@msu.edu; 6 Kent County Intermediate School District (Michigan), Grand Rapids, MI 49525, USA; 7 Health Department of Northwest Michigan, Charlevoix, MI 49720, USA

**Keywords:** community engagement, development, nonpharmacological, pediatric, pain management, rural, cognitive behavioral

## Abstract

Background/Objectives: This paper details the development of the Helping Educators Learn Pediatric Pain Assessment and Intervention Needs (HELP PAIN) program. Methods: HELP PAIN is an 8 h live training program for school providers (e.g., school nurses and social workers) to use evidence-based nonpharmacologic pediatric pain management tools. The program’s scope reflected the reach of the participating intermediary community organizations, resulting in focused training in rural northwest Michigan due to the Health Department of Northwest Michigan’s service in that region and statewide reach due to the broad representation of members from the Michigan Association of School Nurses. Results: We describe the development of the HELP PAIN program, drawing on evidence-based nonpharmacologic (e.g., cognitive behavioral and mindfulness meditation) strategies for pediatric pain management. Conclusions: In partnership with the key community organizations and community partners, we developed, interactively refined, and delivered this training program.

## 1. Introduction

Pediatric chronic pain is a common problem affecting 10% or more of school-aged children [1]; it can result in poor academic performance [2,3], social problems [4], and mental health problems [5,6] (e.g., anxiety, depression, and PTSD) and increases the risk of substance use disorders in adulthood [7]. Unfortunately, children with chronic pain are absent for an average of 22% of all school days [8] and are at increased risk of failure to graduate from high school [9]. The impact of chronic pain in children is especially evident in rural areas and other communities [10,11], where there is limited access to health and behavioral health providers.

Schools may be the optimal setting to address child pain complaints, as school providers, including school nurses or other allied professionals (e.g., social workers, teachers, and other unlicensed support staff, such as office assistants or healthcare workers), are often the frontline individuals tasked with addressing child pain. Indeed, pain issues (e.g., abdominal pain and headaches) are among the most common presented complaints in the school nurse’s office, accounting for more than one-third of visits [12]; however, school nurses report limited knowledge of the causes of pediatric chronic pain and effective treatments [13]. There may not even be a school nurse present in some schools, including those in rural communities. Therefore, the type of staff member addressing pain may vary between different schools. Unfortunately, knowledge of appropriate approaches to the management of pediatric pain in school settings across disciplines is limited [14].

Cognitive behavioral strategies for pain management are evidence-based approaches that can be effective for managing pediatric pain conditions and aim to improve coping and restore functioning to manage pain [15,16]. Mindfulness meditation techniques can also be helpful for the management of pediatric chronic pain conditions [17] and are increasingly integrated into cognitive behavioral programs for pediatric chronic pain [18]. Pain-focused coping skills programs such as these are traditionally delivered by a pediatric pain psychologist with specialized training [19]. Unfortunately, behavioral health providers (including those who specialize in pain management) are unavailable in many areas. In Michigan, the gap in access to behavioral care is limited in rural areas [20].

Psychologists and mental health specialists need not be the sole deliverers of pain-focused coping skills programs for school-aged children. With proper training and support, nurses [21] and other health professionals [22] can deliver these programs. Moreover, mental health specialists in school settings who may already be trained in cognitive behavioral strategies for managing pediatric mental health concerns [23] can be trained in applying their foundational knowledge of cognitive and behavioral strategies to pediatric pain management. School nurses, however, do not typically receive training in nonpharmacologic pediatric pain management strategies, despite their care of school-aged children with pain, the evidence of training success [21], and the clear potential for benefit to the students they serve. Thus, either as part of a multi-disciplinary team or in a champion role, a variety of school providers have the potential to positively impact child pain outcomes.

This paper outlines the process of development of the Helping Educators Learn Pediatric Pain Assessment and Intervention Needs (HELP PAIN) program. HELP PAIN is a training program for multi-disciplinary school providers, including school nurses and social workers, on the use of nonpharmacologic pediatric pain management techniques. HELP PAIN specifically trains school providers to use evidence-based cognitive behavioral and mindfulness meditation strategies for pediatric pain management. The development of the training program occurred in collaboration with community stakeholders and school providers who would implement the strategies with students in their schools [24]. Several key organizational partnerships (as detailed below), one with reach across the state and another in northwest Michigan, allowed us to take an approach to outreach/training that was both broad and focused. Approaches that involve and lean on the expertise of local community members are essential to the success of community initiatives and are fundamental to community psychology and community-engaged research, which aims to improve the quality of life of individuals via a supportive “community” network that can be influenced by social, political, and economic factors [24,25,26].

## 2. Methods

**Figure 1:**
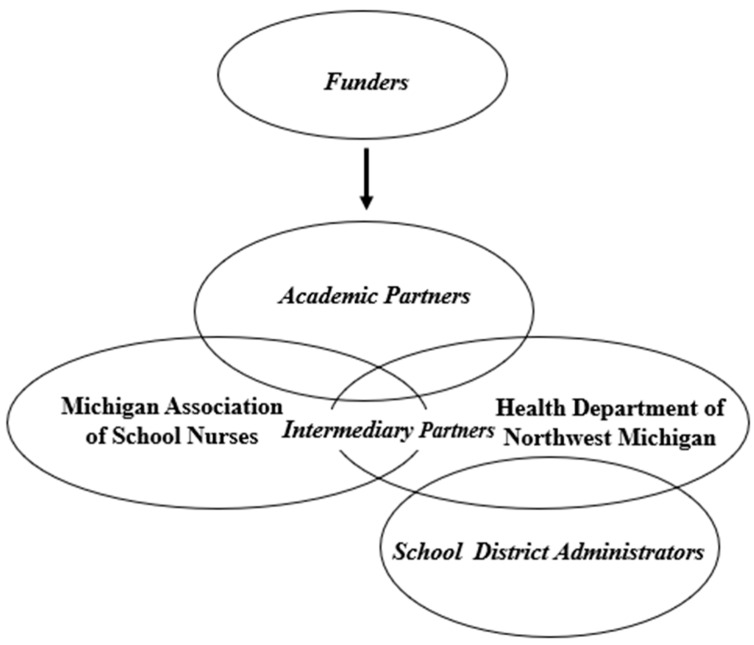
HELP PAIN Development Team. This project involved the collaboration of two community-based organizations, the Michigan Association of School Nurses (MASN) and the Health Department of Northwest Michigan (HDNWM), with the academic partners at Michigan State University (MSU) and Akron Children’s Hospital. The two community-based organizations (MASN and HDNWM) overlapped and interacted through shared members, while each organization worked with academic partners directly and separately. The funders of this project, the Blue Cross Blue Shield Foundation of Michigan and the Michigan Health Endowment Fund, worked with the project leader at MSU to fund this project. In addition, the academic team established partnerships with specific schools (and their district administers) through HDNWM. The school administrators involved in this project were from Ellsworth Community Schools, Concord Academy of Petoskey, and Alanson Public Schools.

Multi-system approach. Our approach to developing and executing HELP PAIN with multiple partners and organizations, as detailed below, is depicted in Figure 1. The Consolidated Framework for Implementation Research (CFIR) was used to identify potential barriers and facilitators to guide our implementation process [27,28]. This paper primarily highlights the HELP PAIN development work conducted in consultation with local teams.

Academic team. Our academic team included a project leader (NRC), a pediatric psychologist at Michigan State University (MSU) with internationally recognized expertise in developing and testing cognitive behavioral approaches to pediatric pain management [18,29]. The project leader initiated the project and (after forming partnerships, as detailed below) developed a draft of the training to share with other scholarly and community-based team members for iterative refinement. A senior health systems researcher (JA), a family medicine physician practicing in the focus region of northwest Michigan (ALW), and a biostatistician (SP), all based at MSU, also collaborated on the project. Additionally, a graduate student of psychology at MSU (MRR) and a practicing pediatric pain psychologist (SCL; based at Akron Children’s Hospital), both with experience using nonpharmacologic coping skills strategies to manage pain in school-aged children, collaborated. Collectively, their roles included review of and feedback on training materials (JA, ALW, SCL, and MRR) and study design (SP). A project assistant (MA) helped with coordinating the project and helped design the display of training materials. We obtained IRB approval at MSU to conduct the research.

Intermediary partners. As access to behavioral healthcare is limited across Michigan, we sought to reach school providers throughout the state. We also conducted focused outreach within northwest Michigan, a rural region of the state with limited access to pediatric behavioral healthcare [30]. We recognized the value of partnering with intermediary organizations to conduct our work as this approach is commonly used in other research areas (e.g., addressing worker health in small businesses) with success [31]. To form such partnerships, we enlisted university colleagues with prior success in collaborating with community organizations to establish initial connections.

The organizational partnerships we formed were with the Health Department of Northwest Michigan (HDNWM), which serves four rural counties (Charlevoix, Emmet, Antrim, and Otsego) in the northwest lower Michigan peninsula. The HDNWM employs both school nurses and mental health professionals, such as school social workers, who serve schools in that region. Secondly, we partnered with leaders from the Michigan Association of School Nurses (MASN), a professional organization for school nurses across the state. Although MASN does not employ its members, the organization supports and connects a large network of school nurses, including by holding professional conferences and training sessions.

First, we conducted preliminary work to build relationships during the 2021–2022 academic year by querying members of these organizations regarding their interest in pediatric pain management training and offering an introductory educational seminar to intermediary organizations consisting of providers already embedded within Michigan schools [32]. This preliminary work with 71 providers from MASN and the HDNWM suggested that the vast majority (89.6%) found the introductory seminar helpful and (87.5%) desired additional training in nonpharmacological pediatric pain management strategies, driven by a shared recognition of the common problem of pediatric pain in schools and the need for more effective solutions.

Funders. Given our shared commitment to improving pediatric chronic pain and the successful preliminary work, which demonstrated a need for such training by the community partners [32,33], the aforementioned team members (both academic team and intermediary organizational partners) collectively applied for and received funding from the Blue Cross Blue Shield Foundation of Michigan (2022–2023) and the Michigan Health Endowment Fund (MHEF; 2022–2024) to develop (the focus of this paper) and test (detailed in a future paper) the HELP PAIN program. One of our funders (MHEF) suggested that our proposal would be strengthened by directly engaging with administrators of schools within the rural northwest Michigan area; so, we sought to establish those connections as well, facilitated by the HDNWM.

School district/administrative partners. With outreach facilitated by HDNWM (who employs providers serving school districts in their region), administrators from three school districts agreed to support the project (Ellsworth Community Schools, Concord Academy of Petoskey, Alanson Public Schools). The schools offered different levels of augmented support/outreach related to the launch of the program: (1) One school administrator hosted a staff meeting prior to the project launch; the project leader was physically invited to this meeting to present information about the program; (2) another school administrator had their HDNWM staff member (who was undergoing the training) present information about the project during a staff meeting; and (3) another school agreed to send an email blast to staff about the project.

## 3. Results

The content of the HELP PAIN training is detailed in Table 1. The program, first drafted by the program leader, consisted of educational strategies and cognitive and behavioral approaches to managing pediatric pain. Specifically, the educational strategies included gate control theory, which is a biopsychosocial model for understanding how thoughts and feelings impact the pain experience [24,25,26]. They also included parent guidelines for managing pediatric pain, which includes limiting excessive pain symptom monitoring while praising the use of adaptive pain-coping strategies and encouraging engagement with medical providers for symptom evaluation and management as needed. The cognitive strategies included changing maladaptive feelings about pain into more positive or realistic thoughts and problem solving. The behavioral strategies included relaxation strategies, such as deep breathing, progressive muscle relaxation, guided imagery, and behavioral activation. Mindfulness meditation (mindful breathing and mindful eating) for pediatric pain management was also taught. Additional content on developmental adaptations/considerations, managing psychological comorbidities, and sleep hygiene was also included in the program, sometimes in response to partner feedback, as detailed below. The training content was primarily developed to be delivered live (in person or hybrid/virtual) with PowerPoint slides, utilizing graphic design strategies with the goal of creating engaging professional presentations [34]. We also developed a companion HELP PAIN workbook with handouts and tools for trainees; the workbook was available virtually. Educational videos were also created to model the use of skills and strategies for providers, with potential applications for student use. Nurses and social workers were able to receive continuing educational credits for their participation, and MASN members were able to receive modest compensation for participating in the research aspect of the program (e.g., by completing self-report measures and qualitative interviews).

Prior to the initial launch of the program, we solicited feedback from leaders of MASN and HDNWM, the intermediary organizations, and the academic partners. These members provided instrumental feedback on the draft training, which was subsequently revised. The following refinements were suggested and incorporated by members of our academic team: addition of content to facilitate communication with families about medical evaluations for pain; adding educational content tailored for different pain types; and augmented content on gate control theory, school accommodations, and sleep hygiene. Additionally, we originally had a different name for this program (NO PAIN); but one of our funders astutely noted that as we stated in our grant application (and aligned with the evidence [35]), the goal was functional restoration and not necessarily pain elimination. Thus, we changed the name of the program to HELP PAIN.

The MASN leaders also suggested content changes related to sleep hygiene, explaining that trainees should be taught to ask students directly about their sleep environment. They also suggested the integration of an abbreviated training/support for other school professionals, such as unlicensed assistive personnel or healthcare workers who may be tasked with managing student pain, especially in settings where school nurses were not always available. They also suggested pain screening adaptations, such as the use of face pain scale versus a numeric rating scale for younger school-aged children. MASN leaders also recommended tailored/extended consultation/support for nurses outside of the formal training and ways to collect and track data for nurses who train others versus those nurses who provide direct student care. HDNWM and MASN both advocated for optimal delivery approaches tailored to their organization.

The MASN leaders requested a full day of in-person/hybrid training in the fall of 2022 as part of a pre-conference workshop. The HDNWM leaders requested that the content be divided into four 2 h training sessions, delivered primarily in person at required staff meetings throughout the 2022–2023 academic year. Both partner organizations also suggested virtual options that were implemented into the program as hybrid programing for those that could not make the in-person training, individual training for those unavailable for scheduled sessions, and asynchronous delivery options. Refresher (or introductory) mini-courses reviewing the program content were also proposed, both for those who did participate in the comprehensive program to refresh their knowledge and for the new members/those who did not participate in the comprehensive program.

Based on the qualitative feedback after the course and the post-training survey data collected from attendees after the refresher/introductory course (see Table 1), the HELP PAIN content was extremely well accepted by academic partners and leaders/trainees from intermediary organizations. From the qualitative feedback following the HELP PAIN training, one MASN school nurse reported after participating in the training program that they thought “it was really well executed”, and another noted that the slides were generating “a lot of good conversation”. An HDNWM trainee commented that the “slideshow was amazing. It really just mapped it out”. A third MASN participant complimented the program on its ability to expand on skills they felt school nurses already had by giving them the “extra step and extra tools to go a little bit deeper and keep doing those things” to support students. From the refresher course survey, when asked on a Likert scale how much they agreed with the following: “I have received helpful training/education on cognitive behavioral therapy and mindfulness for pain today”, nearly all (127 out of 128 respondents) responded with strongly agree or agree.

After the trainees received HELP PAIN, we solicited additional feedback and modified the program iteratively. For this paper, we detailed the feedback specific to the changes made in the HELP PAIN programming in Table 1. This led to the inclusion of a brief provider screener, rather than relying exclusively on a child report measure, to assess and track child functioning. It also led to the development of new content, including an educational video on how to tailor strategies for neurodivergent school-aged children, and the creation of a handout, which was based on school nurses’ management of asthma, to support a stepped approach to supporting school-aged children in coping with pain. Additional results of the broader trainee feedback on the program feasibility (beyond program format/content) based on qualitative interviews will be available in a future publication.

The evolution of HELP PAIN and community champions. Several champions, who were part of the intermediary organizations and received training, prompted further development of the HELP PAIN program to include additional outreach within their schools. This included hosting broad school staff professional development days about HELP PAIN with some brief demonstrations of its content, including relaxation training and mindful eating, which in one school district was collaboratively run by the project leader and a MASN member/district nurse (KT) who became a champion of the work. These members also co-presented sessions about HELP PAIN during the 2024 MASN spring conference in addition to collaborating on a subsequent grant submission to expand upon the work (as detailed below).

Furthermore, another trainee who became a community champion, an HDNWM school nurse (JP), reached out with an interest in creating and running a pain-coping group based on the HELP PAIN materials. The project leader collaboratively developed a scripted manual and handouts. A small HELP PAIN group was run at that nurse’s school in the spring of 2023, with the HDNWM school nurse and a school social worker co-leader. The project leader also provided ongoing support/consultation (e.g., meetings every other week) for the school nurse during this period.

In addition, the project leader, often in combination with other HELP PAIN team members, participated in additional outreach/engagement activities, including presentations to regional, national, and international audiences. This led to one additional trainee, not associated with MASN or HDNWM, who provided program feedback that led to substantive changes, as detailed in Table 1.

Further program evolution. As noted above, the team also created a HELP PAIN refresher/introductory course. This course was four hours in total and was delivered live via a monthly videoconference during spring 2024. The refresher was created so that it was appropriate for both prior trainees and new staff members, with continuing education credit available for nurses. After the attendees completed the refresher programs, we solicited their comments about the program during a post-training survey. We asked the participants to indicate additional pediatric pain-related content they would like covered in future sessions. They responded with “helping non-verbal/special needs students with pain”. Asynchronous and credit-bearing HELP PAIN online courses for nurses and social workers also are under development in partnership with the MSU School of Social Work and the MSU College of Nursing and are set to be launched during the 2024–2025 academic school year. We also developed print copies of the training materials in response to the community feedback.

The project leader and members of the study team recently applied for and received another grant to augment this work by addressing trauma and substance misuse in addition to pediatric chronic pain symptoms, which is responsive to the grant funders’ call and also to MASN’s feedback to supplement the program by addressing these commonly overlapping concerns. This project will include an expanded team of community partners and plans for enhanced roles for prior community champions/intermediary organizations.

## 4. Discussion

This paper details the development of the HELP PAIN program. To ensure success, it was critically important that the development process included organizations and school providers who serve school-aged children experiencing pain complaints. We sought to create a program that would augment care across the state of Michigan and focused efforts in rural northwest Michigan given the lack of access to available care providers in this region [30]. HELP PAIN was developed to achieve more equitable care for school-aged children with chronic pain [36].

Our HELP PAIN program development process required the involvement of multiple systems/organizations and encompassed team members with different types of expertise. These partners included traditional scholars based in academic settings and the “boots on the ground” experts—those community providers (i.e., school nurses and mental health professionals) and their community organizations already serving school-aged children with chronic pain in school settings. The success of programs such as HELP PAIN is contingent on the community providers voicing the need for such programs [29,30], engaging in training, and implementing program strategies over time. Therefore, we engaged leaders from the partner organizations and the trainees to iteratively develop the program. Our ongoing development process was the first step towards creating a successful program to achieve more equitable and direct access to pediatric behavioral health care for child pain complaints in schools.

It is important to note that our efforts began not just at the launch of this project, but were predated by a critical period of relationship building between the academic team, the community partners, and the intermediary organizations and their members, which included preliminary outreach/education and assessing community interest and needs [32,33]. This was of the utmost importance because the academic team members (with the exception of a family medicine physician located in northwest Michigan) were outsiders to the communities of focus. When devising solutions for specific communities, such as those in rural northwest Michigan, a cultural adaptation model, which includes information gathering, consultation with a local team, and external validation, offers a helpful framework to guide program development and adaptations for specific communities [37].

Indeed, we found that taking the time to gauge the interest of community members, including both leaders and members of our partner organizations, led to meaningful collaborative work. For example, the HELP PAIN training content and format, while guided by the project leader’s expertise, required community input to ensure relevant content and appropriate delivery of the program to best meet community needs. Examples of this include restructuring the training format to be flexibly delivered, such as through offering both full-day training and segmented training over an academic year, depending on organizational partner preferences. While the project leader encouraged in-person training opportunities, hybrid/virtual training opportunities were also incorporated into the program to increase accessibility. Future asynchronous courses are planned (with professional educational credits available) to increase and continue program uptake.

We note that some partner preferences may vary and need to be honored. For example, when working with the school district administrators, some welcomed in-person engagement with the academic team while others preferred that their own embedded providers promote the project, as opposed to the outside academic expert(s). It was important that such partners were given options and support with regard to preferences for levels of engagement with the academic team. This builds trust and may facilitate increasing levels of collaboration and engagement over time.

Indeed, the community engagement in the research model showed different levels of community engagement based on the number of stakeholders [38]. We incorporated community members across different levels through interviewing/surveying trainees (lower levels) and engaging a team of community research partners (higher level) to make collaborative research decisions. Although we did not engage community members in PI/co-PI roles for the development of HELP PAIN, it should be noted that collaborative community-engaged research roles are not static and can evolve as partnerships develop over time and trust between members continues to build [39]. Academic researchers can work towards serving the community’s needs and building trust. Community members may then in turn take on more of what is commonly considered “traditional” research tasks, such as identifying needs, generating research questions, data analysis, and writing/presenting scholarly outputs. Indeed, some of our community team partners have taken on increasingly greater roles in program development, such as spearheading the development of group-based approaches to care, co-presenting findings, launching professional development days, tailoring training content, and collaborating on grant submissions.

This program was designed to primarily address the needs of school-aged children who are in school settings with moderate symptoms of pain and related concerns. We also designed HELP PAIN to provide some guidance about meeting the needs of school-aged children with more severe symptomatology (e.g., how to address school-aged children with severe mental health concerns AND chronic pain), which may involve prioritizing mental health concerns and utilizing outside referrals. We acknowledge that working with a medical provider for evaluation and symptom management may also be part of a comprehensive care plan. Our iterative modifications of HELP PAIN content based on trainee feedback also suggested a need for additional tools to better support neurodivergent school-aged children and other groups with complex needs. The limitations include partnership with just two intermediary organizations located within the state of Michigan, which may contribute to relatively homogenous community partner feedback. Future programming should continue to address the needs of diverse community organization groups, expanding the focus across the state and country.

Ultimately, it is important that the content fits the learning needs of the community and addresses the problems they see. Pediatric chronic pain is an issue for which providers would like support in managing, but they note that pain and other problems (e.g., substance abuse risk and traumatic distress) may co-occur and place vulnerable school-aged children at increased risk. Indeed, the literature suggests that rates of traumatic distress are higher in school-aged children with chronic pain and are associated with poorer outcomes [40,41,42]. Furthermore, chronic pain in adolescents is associated with opioid misuse in adulthood, with trauma and substance misuse increasing this risk [7]. To be responsive to community member needs, future programming related to HELP PAIN will augment training in strategies to better address these issues that commonly impact school-aged children with chronic pain.

HELP PAIN was developed with sustainability in mind. Since this program is intended for the training of school providers, those providers, and the organizations they were part of, were included as the community partners in the training development phase. Future work testing the impact of such a program on pediatric outcomes in community settings will be important.

The collaborative development of HELP PAIN aims to support community needs. Further testing and evaluation of the implementation outcomes is underway. Additionally, the evolution of this program, both the content and the roles of the research/community team members driving this work, will be critically important to promote future success and develop a program applicable and relevant to the communities it is intended to serve.

## 5. Conclusions

In conclusion, the HELP PAIN training program was developed and refined in collaboration with academic partners, school partners, and community organizations. These partnerships were utilized to maximize the program’s potential in successfully training school professionals in evidence-based nonpharmacologic strategies to better support school-aged children with chronic pain.

## Figures and Tables

**Table 1:** HELP PAIN program content.

		Modifications	
		Academic Partners ^1^	Community Partners ^2^
Content	Topic		
General		Implemented overview slide Added “practice time” transition slides to clarify when practice would be occurring	The database was updated to include a survey that asks if “providers train others or share the training” (MASN ^3^) Hybrid delivery (MASN ^3^, HDNMW ^3^) Survey questions to determine whether the training was in person, virtual, or hybrid (MASN ^3^) The delivery format differed for the two community organizations. One 8-hour session (MASN ^3^) Four 2-hour sessions (HDNMW ^3^) Added 3-item provider assessment of functioning tool and improvement following HELP PAIN in response to the impracticality of using child-reported measures of functional impairment (MASN ^4^, HDNWM ^4^) Created professional development day materials for training of additional school staff (MASN ^4^) Created group-based program for school school-aged children (HDNWM ^4^) Created intro refresher series (MASN ^4^) Paper copies of toolkit created. Asynchronous credit-bearing modules in development (MASN ^4^)
Chronic Pain Education		Added explanation of chronic versus acute pain	Added that a challenge facing schools is not having school nurses or other medical staff to address pain (MASN ^3^)
Using Screening Tools	Functional Disability Inventory Assessing Pain School Attendance/Nurse Visits Face Pain Scales	Augmented information on encouraging families to pursue medical evaluations of symptoms if not previously carried out	Added face pain scales (MASN ^3^)
Foundational Skills:			
School Accommodations	Accommodation Guidelines	Created a “fast pass” to include in toolkit for kids to use in schools for pain ^5^	
Educational Strategies	- Gate Control Theory of Pain * - Parent Guidelines *	Added stress, muscle tension, thinking about pain, people asking about pain, under-activity/overactivity, sleeping too much/too little Added imagery of gate control theory of pain Added “Sick Day Rules” in parent guidelines	
Behavioral Strategies	- Deep Breathing * - Mindful Breathing * - Progressive Muscle Relaxation * - Guided Imagery * - Biofeedback - Problem Solving * - Pleasant Activities * - Activity Pacing *		
Advanced Skills: Tailoring the Program	Developmental Considerations Stepped Care	Created a “pain action plan” which outlines strategies depending on the level of pain, similar to an “asthma action plan” ^5^	Created video content on tailoring skills for neurodivergent school-aged children * (refresher course participant ^4^)
Addressing comorbid mental health issues			Future content underway to address trauma/risk of substance use disorders (MASN ^4^)
Assertiveness Training	Being Assertive *		
Sleep Hygiene		Added sleep hygiene checklist to toolkit for students to mark which aspects they have trouble with and which they would like to focus on improving	Added assessment of living situation, including whether the child has access to private sleep space (MASN ^3^)
Mindfulness	Mindful Eating * Surfing the Wave 5 Senses Exercise Self-Compassion	Added “mindful walking, 5 senses exercise, 5-4-3-2-1 exercise”	Adjustment to mindful eating script for younger school-aged children (MASN ^4^)
Future Planning			

Funders also influenced aspects of the program, including the name of program and future plans to address trauma and substance use. ^1^ The academic partners for this project include members of Michigan State University and Akron Children’s Hospital. ^2^ The community partners for this project are the Michigan Association of School Nurses and The Health Department of Northwest Michigan. ^3^ Leadership from the organization. ^4^ Trainee from the organization. ^5^ PhD student nurse trainee, who elected to complete training after attending an international research presentation about the program by the HELP PAIN project leader, suggested the modification. They are not members of the community partner organizations. * A video is available on this topic at the HELP Lab YouTube Channel.

## Data Availability

The original contributions presented in the study are included in the article, further inquiries can be directed to the corresponding author.

## References

[1] King S., Chambers C.T., Huguet A., MacNevin R.C., McGrath P.J., Parker L., MacDonald A.J. (2011). The epidemiology of chronic pain in children and adolescents revisited: A systematic review. Pain.

[2] Groenewald C.B., Tham S.W., Palermo T.M. (2020). Impaired school functioning in children with chronic pain: A national perspective. Clin. J. Pain.

[3] Logan D.E., Simons L.E., Stein M.J., Chastain L. (2008). School impairment in adolescents with chronic pain. J. Pain.

[4] Gibler R.C., Beckmann E.A., Lynch-Jordan A.M., Kashikar-Zuck S., Mano K.E.J. (2019). Characterizing social and academic aspects of school anxiety in pediatric chronic pain. Clin. J. Pain.

[5] Vinall J., Pavlova M., Asmundson G.J., Rasic N., Noel M. (2016). Mental health comorbidities in pediatric chronic pain: A narrative review of epidemiology, models, neurobiological mechanisms and treatment. Children.

[6] Shelby G.D., Shirkey K.C., Sherman A.L., Beck J.E., Haman K., Shears A.R., Horst S.N., Smith C.A., Garber J., Walker L.S. (2013). Functional abdominal pain in childhood and long-term vulnerability to anxiety disorders. JAMA Pediatr..

[7] Groenewald C.B., Law E.F., Fisher E., Beals-Erickson S.E., Palermo T.M. (2019). Associations between adolescent chronic pain and prescription opioid misuse in adulthood. J. Pain.

[8] Norton J., Southon N. (2020). Exploring the prevalence of pediatric chronic pain and school absenteeism for therapists working in schools: A systematic review with meta-analysis. Phys. Occup. Ther. Pediatr..

[9] Murray C.B., Groenewald C.B., de la Vega R., Palermo T.M. (2020). Long-term impact of adolescent chronic pain on young adult educational, vocational, and social outcomes. Pain.

[10] Boey C.C.M., Yap S., Goh K.L. (2000). The prevalence of recurrent abdominal pain in 11-to 16-year-old Malaysian schoolchildren. J. Paediatr. Child Health.

[11] Tripp D.A., VanDenKerkhof E.G., McAlister M. (2006). Prevalence and determinants of pain and pain-related disability in urban and rural settings in southeastern Ontario. Pain Res. Manag..

[12] Schneider M.B., Friedman S.B., Fisher M. (1995). Stated and unstated reasons for visiting a high school nurse’s office. J. Adolesc. Health.

[13] Youssef N.N., Murphy T.G., Schuckalo S., Intile C., Rosh J. (2007). School nurse knowledge and perceptions of recurrent abdominal pain: Opportunity for therapeutic alliance?. Clin. Pediatr..

[14] Logan D.E., Catanese S.P., Coakley R.M., Scharff L. (2007). Chronic pain in the classroom: Teachers’ attributions about the causes of chronic pain. J. Sch. Health.

[15] Fisher E., Villanueva G., Henschke N., Nevitt S.J., Zempsky W., Probyn K., Buckley B., Cooper T.E., Sethna N., Eccleston C. (2022). Efficacy and safety of pharmacological, physical, and psychological interventions for the management of chronic pain in children: A WHO systematic review and meta-analysis. Pain.

[16] Fisher E., Law E., Dudeney J., Palermo T.M., Stewart G., Eccleston C. (2018). Psychological therapies for the management of chronic and recurrent pain in children and adolescents. Cochrane Database Syst. Rev..

[17] Ali A., Weiss T.R., Dutton A., McKee D., Jones K.D., Kashikar-Zuck S., Silverman W.K., Shapiro E.D. (2017). Mindfulness-based stress reduction for adolescents with functional somatic syndromes: A pilot cohort study. J. Pediatr..

[18] Cunningham N.R., Nelson S., Jagpal A., Moorman E., Farrell M., Pentiuk S., Kashikar-Zuck S. (2018). Development of the Aim to Decrease Anxiety and Pain Treatment for Pediatric Functional Abdominal Pain Disorders. J. Pediatr. Gastroenterol. Nutr..

[19] Nelson S., Coakley R. (2018). The pivotal role of pediatric psychology in chronic pain: Opportunities for informing and promoting new research and intervention in a shifting healthcare landscape. Curr. Pain Headache Rep..

[20] State of Michigan Behavioral Health Access Study. https://mihealthfund.org/wp-content/uploads/2022/12/Health-Fund-2022-Behavior-Health-Access-Study-1.pdf.

[21] Dalton J.A., Keefe F.J., Carlson J., Youngblood R. (2004). Tailoring cognitive-behavioral treatment for cancer pain. Pain Manag. Nurs..

[22] Dworkin S.F., Huggins K.H., Wilson L., Mancl L., Turner J., Massoth D., LeResche L., Truelove E. (2002). A randomized clinical trial using research diagnostic criteria for temporomandibular disorders-axis II to target clinic cases for a tailored self-care TMD treatment program. J. Orofac. Pain.

[23] Choi S.Y., Rusch A., Koschmann E., Bilek E.L., Lane A., Abelson J.L., Eisenberg D., Himle J.A., Fitzgerald K.D., Liebrecht C. (2022). How Effective Are School Professionals at Identifying Students Who Might Benefit from Cognitive Behavioral Therapy? Baseline Data from the Adaptive School-Based Implementation of Cognitive Behavioral Therapy Trial. Proceedings of the Frontiers in Education.

[24] Marks D.F. (2002). Freedom, Responsibility and Power: Contrasting Approaches to Health Psychology.

[25] Sarason S.B. (1974). The Psychological Sense of Community: Prospects for a Community Psychology.

[26] Murray M., Nelson G., Poland B., Maticka-Tyndale E., Ferris L. (2004). Assumptions and values of community health psychology. J. Health Psychol..

[27] Damschroder L.J., Lowery J.C. (2013). Evaluation of a large-scale weight management program using the consolidated framework for implementation research (CFIR). Implement. Sci..

[28] Damschroder L.J., Reardon C.M., Widerquist M.A.O., Lowery J. (2022). The updated Consolidated Framework for Implementation Research based on user feedback. Implement. Sci..

[29] Cunningham N.R., Kalomiris A., Peugh J., Farrell M., Pentiuk S., Mallon D., Le C., Moorman E., Fussner L., Dutta R.A. (2021). Cognitive behavior therapy tailored to anxiety symptoms improves pediatric functional abdominal pain outcomes: A randomized clinical trial. J. Pediatr..

[30] Universities of Wisconsin Population Health Institute Michigan—Mental Health Providers. www.countyhealthrankings.org.

[31] Cunningham T.R., Sinclair R. (2015). Application of a model for delivering occupational safety and health to smaller businesses: Case studies from the US. Saf. Sci..

[32] Siebert E.M., Pierce S.J., Ely S., Cunningham N. (2023). Feasibility and Training Outcomes Following an Introductory Workshop on Pediatric Pain-Focused CBT For School Providers. J. Pain.

[33] Siebert E., Pierce S.J., Ely S.L., Cunningham N.R. (2024). The Need and Impact of a Brief Educational Seminar on Pediatric Pain-focused CBT for School Providers. Clin. J. Pain.

[34] Rivera E. More than PowerPoint. https://www.youtube.com/c/EchoRivera.

[35] Lynch-Jordan A.M., Sil S., Peugh J., Cunningham N., Kashikar-Zuck S., Goldschneider K.R. (2014). Differential changes in functional disability and pain intensity over the course of psychological treatment for children with chronic pain. Pain.

[36] Cunningham N.R., Reid M.R., Love S.C., Connelly M.A. (2023). Commentary: Actionable Steps for Addressing Pediatric Pain in Rural and Underserved Communities: Disrupting Our Approach to Psychological Science and Care. J. Pediatr. Psychol..

[37] Perera C., Salamanca-Sanabria A., Caballero-Bernal J., Feldman L., Hansen M., Bird M., Hansen P., Dinesen C., Wiedemann N., Vallières F. (2020). No implementation without cultural adaptation: A process for culturally adapting low-intensity psychological interventions in humanitarian settings. Confl. Health.

[38] Boyer A.P., Fair A.M., Joosten Y.A., Dolor R.J., Williams N.A., Sherden L., Stallings S., Smoot D.T., Wilkins C.H. (2018). A multilevel approach to stakeholder engagement in the formulation of a clinical data research network. Med Care.

[39] Doberneck D.M., Dann S.L. (2019). The degree of collaboration abacus tool. J. High. Educ. Outreach Engagem..

[40] Nelson S., Simons L.E., Logan D. (2018). The incidence of adverse childhood experiences (ACEs) and their association with pain-related and psychosocial impairment in youth with chronic pain. Clin. J. Pain.

[41] Nelson S., Cunningham N., Peugh J., Jagpal A., Arnold L.M., Lynch-Jordan A., Kashikar-Zuck S. (2017). Clinical profiles of young adults with juvenile-onset fibromyalgia with and without a history of trauma. Arthritis Care Res..

[42] Nelson S., Cunningham N. (2020). The Impact of Posttraumatic Stress Disorder on Clinical Presentation and Psychosocial Treatment Response in Youth with Functional Abdominal Pain Disorders: An Exploratory Study. Children.

